# Assisted Reproductive Techniques and Risk of Congenital Heart Diseases in Children: a Systematic Review and Meta-analysis

**DOI:** 10.1007/s43032-023-01252-6

**Published:** 2023-05-05

**Authors:** Giuseppe Gullo, Marco Scaglione, Antonio Simone Laganà, Antonio Perino, Alessandra Andrisani, Vito Chiantera, Gaspare Cucinella, Georgios Gitas, Fabio Barra, Gaetano Riemma

**Affiliations:** 1 Department of Obstetrics and Gynecology, Villa Sofia Cervello Hospital, IVF Unit, University of Palermo, Palermo, Italy; 2 Department of Neuroscience, Rehabilitation, Ophthalmology, Genetics and Maternal-Child Sciences, University of Genoa, Genoa, Italy; 3 Unit of Gynecologic Oncology, ARNAS “Civico–Di Cristina–Benfratelli,” Department of Health Promotion, Mother and Child Care, Internal Medicine and Medical Specialties (PROMISE), University of Palermo, Palermo, Italy; 4 Department of Women’s and Children’s Health, Gynaecologic and Obstetrics Clinic, University of Padua, Padua, Italy; 5 Department of Gynecology, University Hospital Charite, Campus Mitte, Berlin, Germany; 6 Department of Neurosciences, Rehabilitation, Ophthalmology, Genetics, and Maternal and Child Health (DINOGMI), University of Genoa, IRCCS Ospedale Policlinico San Martino, Genoa, Italy; 7 Department of Woman, Child and General and Specialized Surgery, Obstetrics and Gynecology Unit, University of Campania “Luigi Vanvitelli”, Largo Madonna delle Grazie 1, 80138 Naples, Italy

**Keywords:** Congenital heart disease, In vitro fertilization, IVF, Assisted reproductive techniques, Cardiac defects

## Abstract

Infertility is a growing phenomenon and leads to an increased use of assisted reproductive techniques (ARTs). In recent years, concerns about the safety of these procedures emerged and ARTs were hypothesized to be a risk factor for developing congenital heart diseases (CHDs) in offspring. Our aim is to investigate the association between ART and CHD, specifying results according to various subtypes of defects. We performed a systematic review and random-effects meta-analysis following the PRISMA guidelines. MEDLINE and Google Scholar were searched from January 2011 to May 2022. Data about incidence of CHD in ART were tabulated and extracted from all the studies included. Twenty*-*four studies were included. Pooled incidence of CHDs after IVF pregnancies was 3% (95% CI 0.3–0.4; *I*^2^ = 99%), decreasing to 1% (95% CI 0.00–0.01; *I*^2^ = 93%) for major CHDs only. An increased risk of CHDs, especially minor (i.e., not requiring surgical correction), seems to occur in ART compared with non-ART pregnancies [RR 1.71 (95% CI 1.25–2.34; *I*^2^ = 99%)]. For major CHDs, not enough evidence is available to assess the real risk. Moreover, some confounding factors (i.e., maternal age and male infertility) seem to play a critical role to determine an increased risk of CHDs. Conflicting results emerged among the studies, setting the need for further research to validate the actual evidence and state the real risk of CHD following ART pregnancies.

**Supplementary Information:**

The online version contains supplementary material available at 10.1007/s43032-023-01252-6.

## Introduction

The frequency of congenital heart diseases (CHDs) in the general population is estimated to be around 1% [1]. However, differences in definition, population characteristics, and diagnostic method lead to a wide range of prevalence varying between 4 and 50 cases per 1000 live births [2]. CHDs are commonly classified into cyanotic (tetralogy of Fallot (TOF), transposition of the great arteries, tricuspid atresia, pulmonary atresia, truncus arteriosus persistence, total anomalous pulmonary venous return) and non-cyanotic. These are further divided into left-to-right-shunt pathologies (ventricular septal defects, patent ductus arteriosus, and atrial septal defects) and outflow obstruction pathologies (pulmonary stenosis, aortic stenosis, coarctation of aorta). Among CHDs, interventricular defects are certainly the most frequent (around 35%), followed by interatrial defects (around 7%). The need for surgical intervention on non-cyanotic CHDs is variable and depends on the severity of the lesion, e.g., small septal defects may not necessarily be clinically significant enough to necessitate correction. In contrast, most cyanotic CHDs require surgical correction. For example, TOF (about 5% of CHDs) requires different types of total surgical corrective procedures according to the different subtype. There are some conditions, such as hypoplastic left heart syndrome and pulmonary atresia with intact ventricular septum, in which multistage surgical correction is required. Of note, the currently existing trans-catheter and surgical techniques to manage cyanotic CHDs are safe, effective, and can be performed at a relatively low risk [3].

The etiology of CHDs is generally unknown and only 15% of cases can be traced back to a known cause, mostly genetically defined syndromes such as Down syndrome, trisomy 13, trisomy 18, Turner syndrome, DiGeorge syndrome, Alagille syndrome, Holt–Oram syndrome, and Noonan syndrome. The causes of non-syndromic congenital defects are more discussed: environmental factors, maternal diabetes mellitus, phenylketonuria, maternal obesity, alcohol use, rubella infection, febrile illnesses, use of certain drugs (for example, thalidomide), exposure to organic solvents or herbicides, maternal age >40 years, and paternal age >35 years are involved. The etiology of the remaining cases of non-syndromic CHDs is multifactorial, suggesting that various genetic and environmental factors interact [2]. The use of assisted reproductive techniques (ARTs) has also been proposed as one of the potential contributory causes of the development of CHDs in newborns. Infertility is a steadily growing phenomenon and now represents a real social problem; the prevalence in recent years has increased uniformly and globally [4].

The increasing prevalence of infertility issues in the general population has led to a growing demand for ART, resulting in increased doubts regarding the health of children born after ART. The aim of this systematic review is to evaluate the correlation between the use of ART and the development of CHDs in newborns. Being a heterogeneous group of pathologies, it is important to take into account how the various subtypes of CHD relate to ART. On this purpose, prevention and/or management protocols could be implemented, if necessary, according to the new emerged data.

## Methods

This systematic review followed the Preferred Reporting Items for Systematic Reviews and Meta-Analyses (PRISMA) guidelines for methodology and data extraction [5], and was registered in PROSPERO (CRD42022383106) before to start the search. The research protocol was created a prior, and it carefully addressed the literature search and reporting, inclusion and examination of articles, and data extraction and statistical analysis.

The studies have been identified through database research on MEDLINE (accessed through PubMed) and Google Scholar. In each database, the following key words were searched for: “assisted reproductive technology and heart defects” and “assisted reproductive technology and congenital heart diseases.” Electronic searches were conducted from January 2011 to May 2022.

No restrictions for geographic location were applied. In addition, the reference lists of all eligible papers were examined to further identify studies not included by electronic searches. The electronic search and the potential eligibility of the qualified studies were independently checked by two authors (A.S.L. and M.S.). Any potential disagreement was resolved by discussion with a third reviewer (A.P.).

This systematic review exclusively included cohort studies, retrospective cohort studies, case-control studies, and cross-sectional studies that investigated the correlation between the use of ART in infertile couples and the development of CHD on subsequent pregnancies. All studies in which the diagnosis of CHD was made by ultrasound, and thus in the gestational period, were included. Reviews, systematic reviews, meta-analyses, and case reports on the subject were excluded from the data collection. Studies that investigated the correlation between ART and congenital malformations in general were also excluded, thus not specifying the results about CHD. Studies published before 2011 were also excluded considering the epidemiological changes in infertility over the last decade. Two authors (F.B. and G.R.) independently extracted data from articles about study characteristics and included populations, methods, and results/outcomes, using a pre-piloted standard form in order to ensure consistency. Any discrepancies were identified and resolved through discussion (with a third external collaborator where necessary).

The quality assessment of included researches was carried out using the criteria outlined in the Newcastle-Ottawa Scale (NOS) [6].

According to these criteria, the judgment of the study is based on three broad elements: the selection of study groups, the comparability of these, and the ascertainment of the outcome of interest. The assessment for the selection of a study involved the following criteria: evaluation of the representativeness of the exposed cohort, selection of the non-exposed cohort, ascertainment of exposure, and demonstration that outcome of interest was not likely to occur spontaneously at the start of the study. The comparability of studies is assessed by evaluating the comparability of cohorts based on the design or analysis. Furthermore, the ascertainment of the exposure is judged upon the methodology for determining the outcome of interest, duration, and adequacy of the follow-up. Adopting the Newcastle-Ottawa Scale criteria, a study can be awarded a maximum of one star for each numbered item within the Selection and Outcome categories. A maximum of two stars can be given for Comparability [6].

Two researchers (A.A. and G.C.) were involved in giving these scores, and any disagreements were resolved by scores given by a third researcher (V.C.).

Meta-analysis was carried out using STATA, version 14.1 (StataCorp., College Station, Texas, USA). The summary measures were reported as risk ratio (RR) or event size proportion (ES) with 95% of confidence interval (CI) after the application of the random-effects model of Der Simonian and Laird. In a conservative approach, the random-effect estimates of ES — depicting the variation of true proportion across included papers — were considered the “main results,” Higgins *I*-squared (*I*^2^) index higher than 0% was employed to assess potential heterogeneity.

## Results

### General Characteristics

**Fig. 1:**
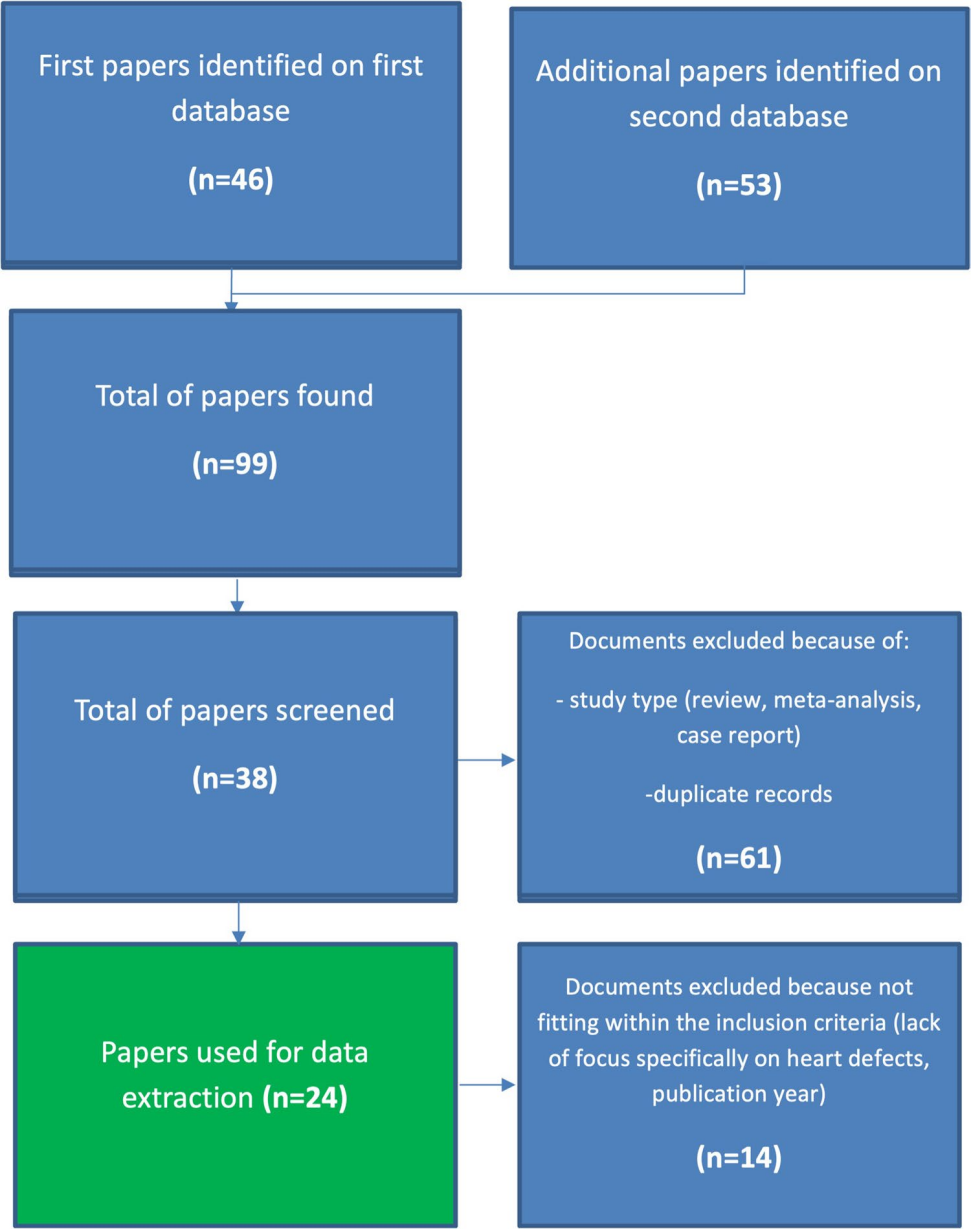
Flow chart for the selection of papers to be included in the review

The first searches in databases showed a total of 99 papers; according to the PRISMA checklist and inclusion criteria, a more accurate identification was performed, selecting 24 papers for data extraction [7–30] (Fig. 1).

The studies analyzed in this systematic review showed a large variety in methodological approaches, including 13 retrospective cohort, four were prospective cohort, four case-control studies, one study was mixed case-control/cohort while another one had a cross-sectional descriptive structure (Table 1).

**Table 1:** Reviewed studies with schematization of the extracted data (reference, author and year, study population, results and quality assessment. *ICSI*, intracytoplasmic sperm injection; *ARTs*, assisted reproductive techniques; *CHDs*, congenital heart diseases; *TOF*, tetralogy of Fallot)

Author (et al.); reference	Year of publication	Type of study	Patients	Results	Quality assessment (NOS score)
Morimoto [17]	2022	Retrospective cohort study	418	Higher proportion of infants conceived by ART in univentricular heart defects, regardless of maternal age and maternal history of miscarriage.	7
Bjorkman [19]	2021	Retrospective cohort study	9252	The odds ratio for CHD in the ART group compared with statewide population rates was not significantly different from baseline population rates (1.4; 95% CI 0.9–2.1).	7
Fauque [22]	2021	Retrospective cohort study	3,501,495	Increased risk of eight malformations in the fresh-ET group compared with the NC group, also heart defects.	6
Galdini [25]	2021	Retrospective cohort study	1511	Increased prevalence of CHD, mainly major defects.	7
Norrman [30]	2021	Retrospective cohort study	7,697,114	After adjustment, there was no significant difference between children born after ART and children born after SC for any cardiovascular disease (adjusted HR [aHR] 1.02; 95% CI 0.86–1.22; *p* = 0.80).	8
Serafin [20]	2021	Cohort study	1581	The chosen method of fertilization or the chosen ovulation method had not a statistically significant effect on the development risk of CHD.	7
Wang [13]	2021	Cohort study	1137	Increased de novo mutations associated with CHD.	8
Zhang [11]	2021	Retrospective cohort study	194,067	Circulatory system malformations were observed to have a non-significant increase in offspring conceived by ART.	8
Aderibigbe [16]	2020	Retrospective cohort study	110	Association with ventricular septal defects (3.6% of all cases in the neonatal period).	6
Wen [14]	2020	Retrospective cohort study	507,390	Not significative association with CHD (OR 1.09; 95% CI 0.93–1.25).	7
Jwa [10]	2019	Retrospective cohort study	59,971	Among ART cycles, male subfertility was associated with significantly greater risks of atrial septal defects (adjusted OR = 3.98, 95% CI 1.12–14.1, *p* = 0.03) compared with fertile men. Oligozoospermia was significantly associated with a greater risk of ventricular septal defects in IVF pregnancies (adjusted OR = 2.68, 95% CI 1.15–6.27, *p* = 0.02).	8
Pavlicek [9]	2019	Cohort study	35,831	Pregnancy after ART (OR 2.8; 95% CI 1.5–5.2) was found to be independent risk factors of CHDs.	7
Patil [27]	2018	Cohort study	363	Increased incidence of mild CHD (2.2%, compared to 1%); similar incidence of severe CHD (1.4% and 1.2%).	7
Shamshirsaz [12]	2018	Cross-sectional descriptive study	14,242,267	As compared with naturally conceiving infants, risk for cyanotic CHD was significantly higher among infants born in ART (adjusted relative risk (aRR) 2.4, 95% CI 2.1 to 2.7).	8
Shechter-Maor [26]	2018	Retrospective cohort study	11,862,780	Association with cyanotic heart defects (OR 2.74, 95% CI 2.42–3.09).	7
Iwashima [21]	2017	Cohort study	2746	Similar prevalence of CHD [4.1% vs. 4.0%]. No significant difference between the groups (*p* = 0.892) about cases of severe CHD.	8
Schofield [7]	2017	Case-control study	894	Logistic regression analysis demonstrated a non-significant increase in the aOR (0.95, 95% confidence interval 0.48–1.88). No significant differences were found for CHD subgroups.	7
Yang [24]	2017	Case-control study	166	There is no difference in NKX2.5 and TBX5 gene mutations between IVF and naturally conceived children with CHD.	7
Panagiotopoulou [29]	2016	Retrospective cohort study	874	In twins, the ART group 8.2% had CHD compared to 4.3% in NC (OR 1.90, 95% CI 1.08–3.34, *p* = 0.024).	6
Heisey [18]	2015	Retrospective cohort study	7120	Association between ART and patent ductus arteriosus	8
Tararbit [23]	2014	Case-control study	4499	Association with TOF after adjustment for confounding factors (adjusted OR 2.6, 95% CI 1.5–4.5). Most (79%) of the effect was a direct effect (i.e., not mediated by multiple pregnancies).	8
Votava-Smith [28]	2014	Retrospective cohort study	2761	The proportion of ART conception was found to be lower in fetuses with CHD (6.9% vs. 10.3%). In a multivariate model controlling for maternal age and multiple gestation, ART was not associated with CHD diagnosis (OR = 1.1 [95% CI 0.77–1.7], *p* = 0.51).	7
Tararbit [8]	2013	Case-control study	317,538	Association with TOF [(OR): 2.4, 95% confidence interval (CI): 1.5–3.7] with the highest risk associated with ICSI (adjusted OR: 3.0, 95% CI: 1.0–8.9).	8
Tararbit [15]	2011	Case-control study	9340	Association with malformations of the outflow tracts and ventriculoarterial connections (adjusted OR 1.7, 95% CI 1.2–2.4) and of cardiac neural crest defects and double outlet right ventricle (adjusted OR 1.7, 95% CI 1.1–2.7).	8

Twelve studies [31–42] might appear to meet the inclusion criteria, but they were finally excluded for year of publication, uncomplete results, and results focused on congenital malformations in children born from ART and not specifically on CHD.

### Quality Assessment

Twenty-two papers had a high methodological quality (more than 7 using NOS criteria), and only 2 studies had a methodological quality lower than the threshold. Some authors expressed the results in terms of odds ratio for the ART-CHD correlation while others compared the incidence of CHD between the group of naturally conceived (NC) children and those born from ART. Some authors reported data only concerning the CHD group in general, and others also specified the report about the various subtypes (Table 1). Table S1 in the supplementary material shows the detailed quality assessment using NOS criteria.

### Meta-analysis

**Fig. 2:**
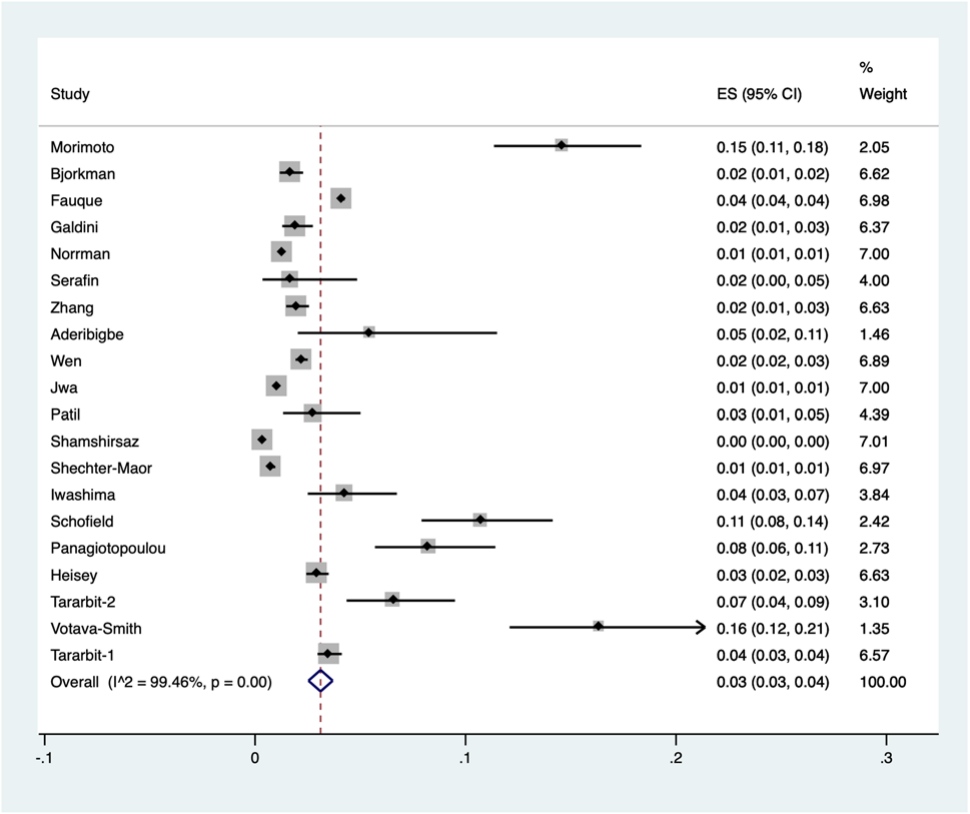
Pooled incidence of congenital heart diseases in fetuses conceived after assisted reproductive techniques

A meta-analysis of proportions was carried out to estimate the overall incidence of CHDs in fetuses conceived after ART. Data were retrieved from 20 studies [7, 8, 10–12, 14, 16–23, 25–30], with 404,972 post-ART pregnancies evaluated. For all the CHDs, a pooled incidence of 3% (95% CI 0.03–0.04; *I*^2^ = 99%) was reported (Fig. 2). Selecting studies that evaluated major CHDs only [12, 21, 26], the pooled incidence decreased to 1% (95% CI 0.00–0.01; *I*^2^ = 93%). Conversely, considering studies that considered minor CHDs only [10, 18, 19, 28] resulted in a pooled incidence of 3% (95% CI 0.02–0.04; *I*^2^ = 97%).

**Fig. 3:**
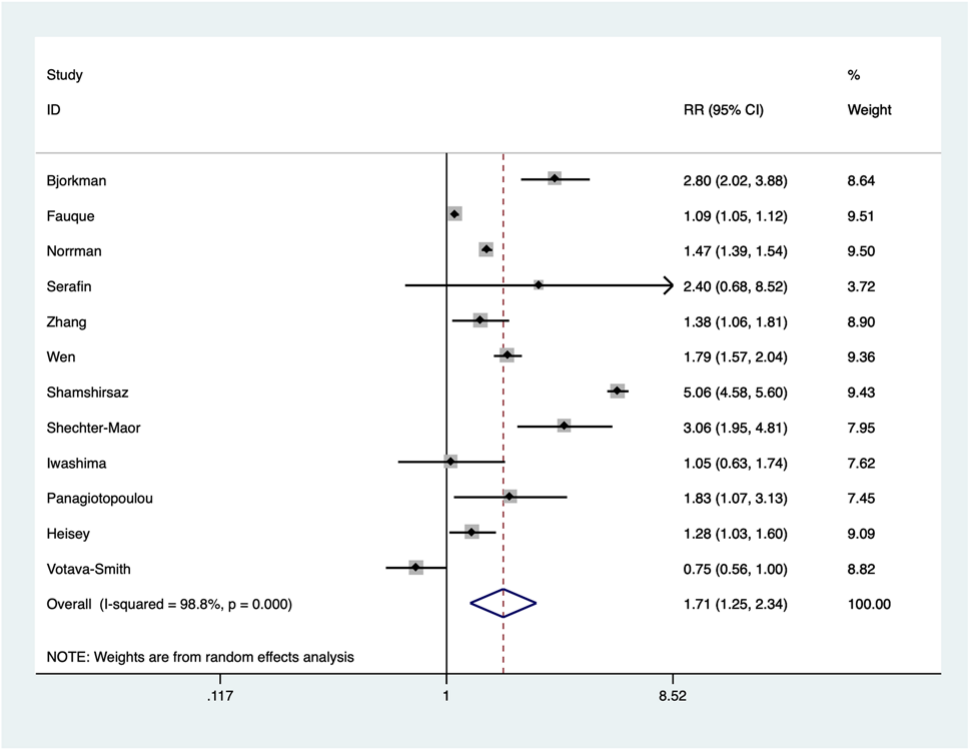
Risk of congenital heart diseases in fetuses conceived after assisted reproductive techniques compared with naturally conceived fetuses

Concerning studies that compared post-ART and naturally conceived fetuses, data about 338,188 post-ART fetuses and 25,909,351 naturally conceived controls were retrieved from 12 papers [11, 12, 14, 18–22, 26, 28–30]. A 1.7-folded increased risk for diagnosing CHDs in ART pregnancies compared with natural conception was noted [RR 1.71 (95% CI 1.25–2.34; *I*^2^ = 99%)] (Fig. 3).

### Synthesis of Results

The principal findings of the studies qualified for systematic review are summarized in Table 1.

Wen et al. [14], in a large retrospective cohort study of 507,390 patients published in 2020, reported a direct association (adjusted OR 1.70; 95% CI 1.48–1.95) between ART and CHD in general (not specifying the subtype). However, after evaluating the mediation of twin pregnancies (87% of the total), the same correlation was not statistically significant (1.09; 95% CI 0.93–1.25) [14].

Patil et al. [27], in a cohort study published in 2018, reported an increased incidence of CHD (1.8% vs. 1%), not specifying the subtype. Differentiating CHD by type, they found that the incidence was higher for non-severe CHD (2.2%, compared to 1%) while, considering severe ones, the incidence was comparable between ART and NC (1.4% and 1.2%) [27].

In contrast, Iwashima et al. [21], in a cohort study published in 2017 on 2746 patients, reported a comparable incidence of CHD between children born from ART and naturally conceived ones (4.1% vs. 4.0%). In agreement with the previous authors, no significant differences between the groups (*p* = 0.892) about cases of severe CHD were found [21].

Considering instead twin pregnancies, Panagiotopoulou et al. [29], in a retrospective cohort study published in 2016, found 8.2% CHD in ART children compared to 4.3% in NC children (OR 1.90, 95% CI 1.08–3.34, *p* = 0.024); nevertheless, the authors did not specify the subtype of heart disease.

Pavlicek et al. [9], in a cohort study published in 2019, found that pregnancy after ART (OR 2.8; 95% CI 1.5–5.2) represents an independent risk factor of CHDs. Also, in this research, the subtype of heart disease was not specified.

Regarding major CHDs, in 2018, in a large retrospective cohort study, Shechter-Maor et al. reported a direct correlation between ART and cyanotic CHDs [26]. Similarly, Galdini et al. [25], in a retrospective cohort study published in 2021 on 1511 patients, reported an association between ART and CHD, mainly in major defects.

Similarly, Taarbit et al. [15], in a cohort study published in 2011, reported an association between ART and malformations of the outflow tracts/ventriculoarterial connections (adjusted OR 1.7, 95% CI 1.2–2.4) and of cardiac neural crest defects and double outlet right ventricle (adjusted OR 1.7, 95% CI 1.1–2.7). The same research group, in a case-control and cohort study published in 2013, reported an association with TOF after adjustment for maternal characteristics, paternal age, and year of birth [(OR): 2.4, 95% CI: 1.5–3.7] with the highest risk associated with intracytoplasmic sperm injection (adjusted OR: 3.0, 95% CI: 1.0–8.9) [8]. For the other subtypes of congenital heart disease, no statistically significant associations were found. However, the authors specified that it is difficult to define whether this relationship is causal or rather mediated by the underlying infertility problems of couples who require ART [8].

The same authors, in a 2014 case-control study, reported an association with TOF after adjustment for maternal and paternal characteristics and year of birth (adjusted OR 2.6, 95% CI 1.5–4.5) [23]. This effect was direct, i.e., not mediated by twin pregnancies, for 79%. Furthermore, intracytoplasmic sperm injection was associated with a 3.5-fold higher odds of TOF (adjusted OR 3.5, 95% CI 1.1–11.2) [23].

Considering major heart diseases, in a recent cohort study published in 2022, Morimoto et al. [17] reported a higher proportion of infants conceived by ART in univentricular defects (16.2%) than in biventricular defects (9.1%) (OR 2.28, 95% CI 1.11–4.68, *p* = 0.025), regardless of maternal age and maternal history of miscarriage.

Shamshirsaz et al. [12], in a large cross-sectional study in 2018, reported that, if compared with naturally conceived infants, risk for cyanotic CHD was significantly higher among infants born in ART (adjusted relative risk (aRR) 2.4, 95% CI 2.1 to 2.7) and non-ART fertility treatment groups (aRR 1.9, 95% CI 1.6 to 2.2).

In contrast, Serafin et al. [20], in a 2021 cohort study on 1581 patients, reported that the method of fertilization or ovulation did not influence the risk of developing CHD in newborns. They also found that paternal infertility itself carries an increased risk of risk mutations for the development of CHD in the offspring [20].

Heisey et al. [18], in a retrospective cohort study published in 2015 on 7120 patients, reported how the only defect specifically associated was ductus arteriosus perviae.

Schofield et al. [7], in a case-control study of 894 patients published in 2017, assessed the impact of confounding factors on ART-CHD correlation. Specifically, a logistic regression analysis demonstrated a non-significant increase in the crude odds for the use of assisted reproduction (OR 1.21, 95% CI 0.66–2.22) in CHD patients [7]. After adjustment for gestation, year of birth, parity, and maternal age, the odds ratio was further reduced (OR 0.95, 95% CI 0.48–1.88). No statistically significant differences were found for the CHD subgroups [7].

Bjkorman et al. [19], in a 2021 retrospective cohort study, analyzed fetal echocardiographies performed in 2230 IVF pregnancies (mean gestational age 22.2 ± 1.4 weeks), most without other known risk factors for CHD. The odds ratio for CHD in the ART group compared with statewide population rates was 1.4 (95% CI 0.9–2.1). In 26 fetuses, CHDs were found; of these, 21 were clinically insignificant ventricular septal defects [19].

Votava-Smith et al. [28], in a cohort study published in 2014 on 2761 patients, showed that the proportion of ART conception was lower in fetuses with CHD (6.9% CHD vs. 10.3% no CHD). Furthermore, in a multivariate model controlling for maternal age and multiple gestation, ART was found to be not associated with CHD diagnosis (OR = 1.1 [95% CI 0.77–1.7], *p* = 0.51), highlighting the role of these confounding factors [28].

Zhang et al. [11], in a large retrospective cohort study published in 2021 on 1967 patients, reported that circulatory system malformations were observed to have a non-significant increase in offspring conceived by ART [11]. Considering malformations in general, it was seen that even weak associations disappear considering multiple pregnancies or mothers >35 years.

Norrman et al. [30], in a larger study published in 2021 on 7,697,114 patients, reported no significant differences between children born after ART and children born after SC for any cardiovascular disease (adjusted HR [aHR] 1.02; 95% CI 0.86–1.22; *p* = 0.80). Congenital cardiomyopathies are also mentioned among these diseases.

In contrast, Fauque et al. [22], in a retrospective cohort study published in 2018, assessed the incidence of 15 types of congenital malformations in children born from fresh embryo-transfer compared to NC ones. A statistically significant increase in heart defects (not considering the subtypes) was found.

Aderibigbe et al. [16], in a retrospective cohort study published in 2020, assessed how the most common cardiac anomaly found in their ART pregnancies was ventricular septal defect, which was identified in 3.6% of all cases in the neonatal period. The authors specified that over 70% of these defects self-resolved after birth.

Jwa et al. [10], in a study published in 2019, reported the incidence of major congenital malformations in a cohort of 59,971 patients. Among ART cycles, male subfertility was associated with significantly greater risks of atrial septal defects (adjusted OR = 3.98, 95% CI 1.12–14.1, *p* = 0.03) compared with fertile men. A further analysis showed that oligozoospermia (i.e., sperm concentrations < 15 million/mL) was significantly associated with a greater risk of ventricular septal defects compared with normal sperm concentrations in ART pregnancies (adjusted OR = 2.68, 95% CI 1.15–6.27, *p* = 0.02) [10].

Regarding de novo mutations associated with the occurrence of a CHD in ART pregnancies, only two studies were found. In a cohort study published in 2021, Wang et al. [13] reported an increased rate of mutations. However, authors specified that paternal infertility itself carries an increased risk of mutations. In contrast, Yang et al. [24], in a case-control study published in 2017, reported no difference in NKX2.5 and TBX5 gene mutations between ART and naturally conceived children with CHD.

## Discussion

The incidence of congenital malformations and of CHDs in children born from ART is a very important topic and deserves a discussion to plan possible diagnostic investigations already during the gestation period.

Analyzing the included studies, different themes emerged. Regarding the incidence of congenital heart disease in children conceived from ART, conflicting results were found. In fact, some authors reported no significant differences [7, 11, 14, 19–21, 30] while the remaining ones reported a significant increase in CHD in children born from ART.

Among them, several authors [9, 22, 29] reported this increase without specifying the subtype of CHD; this aspect is crucial since, as mentioned in the “Introduction,” only major CHDs require surgical correction and consequently should achieve clinical relevance. Thus, although an increase in cardiac malformations in toto was found, such evidence is not useful to plan additional diagnostic tests during gestational age.

Only one author [28] showed a lower proportion of ART conception in fetuses with CHD (6.9% CHD vs. 10.3% no CHD).

Several authors have instead considered the correlation between ART and a specific subtype of congenital heart disease. Even for this aspect, conflicting results emerged from the data extraction.

### Minor CHDs

Specifically, Jwa et al. [10], Patil et al. [27], and Heisey et al. [18] reported statistically significant associations with ventricular septal defects, atrial septal defects, ductus arteriosus, and mild defects in general, respectively. These associations, as mentioned above, concern defects that are not clinically significant as they do not require any therapeutic measures or surgical correction. Specifically, of the ventricular defects prenatally found, about 70% resolve spontaneously after birth should not be related to a proper clinical interest [16].

Similarly, in the study by Bjorkman et al. [19], based on echocardiographic findings, the OR for CHDs in the ART group was not statistically significant (1.4; 95% CI 0.9–2.1) and, of the 26 congenital defects detected, only four were clinically significant. This indicates that 510 fetal echocardiograms would be required for every diagnosis of one clinically significant CHD in the ART group. This raises several doubts on the real benefit of the examination. Indeed, setting the echocardiographic examination as screening in all ART births would lead to increased anxiety and worries in the future parents that might not be justified by a real benefit, given the low clinical impact of the defects that are detected.

The same issue was also raised by Votava-Smith et al. [28]. They reported a lower percentage of ART fetuses with CHD (6.9% vs. 10.3%) and speculated that the discordance of the published data may be related to the fact that pervious ductus arteriosus and isolated interatrial defects were included in the overall amount of CHDs in several studies. These defects may be considered, indeed, para-physiological fetal findings, and therefore, they do not increase the incidence of CHDs in post-ART pregnancies.

### Major CHDs

About major cardiac malformations, i.e., deserving of postnatal surgical correction, authors also reported conflicting results. In particular, Patil et al. [27] and Iwashima et al. [21] reported comparable incidences between ART and NC groups. These data seem to confirm what was highlighted in the previous paragraph about minor CHDs; indeed, according to these authors, the increased incidence of CHD in ART births may not be related to major disorders and therefore not involve any additive diagnostic or therapeutic measures.

On the contrary, other authors have reported an increased incidence of major congenital heart disease in children born on ART. Shamshirsaz et al. [12] and Shechter-Maor et al. [26] reported a statistically significant association with cyanogenic heart disease.

Also regarding major pathologies, the same group of authors [8, 15, 23] found a statistically significant correlation with efflux tract alterations, neural crest migration alteration heart defects, and TOF. However, especially in the study published in 2013, the authors expressed concerns regarding the mediation of pathologies associated with infertility in this correlation; for this reason, they concluded that further future studies are needed to better define this association.

Galdini et al. [25] also reported an increased prevalence of CHD in ART births (1.92%); among the most frequent findings, they reported TOF and hypoplastic left heart syndrome (HLHS), although they did not evaluated the incidence in the general population of these specific types of pathologies. Among the limitations of their study, authors reported the failure to analyze important variables including ethnicity, socioeconomic status, body mass index, smoker status, acid folic and micronutrients intake, and diseases in pregnancy.

As a result of the above, the number of studies focusing on major congenital heart disease is limited and it is therefore not possible to draw a firm conclusion on this issue. However, the discrepancies among the results may be related to the role of confounding factors which deserves an analysis.

### Role of Confounding Factors

Among the authors who have found correlations with severe pathologies such as TOF, doubts emerged regarding the role of all aspects related to the underlying infertility of couples requiring ART. In particular, Taarbit et al. [8], in their 2013 study, emphasized that the association between the risk of TOF and ARTs is doubtful to be considered as a cause-and-effect; in fact the underlying pathological conditions associated with infertility may lead to a higher risk of congenital defects in offspring. However, the same group of authors, in the subsequent case-control study of 2014, reported that the association between ART and TOF remains after adjustment for maternal, paternal characteristics and year of birth. Moreover, authors reported that this association can be considered direct for 79% (mediated therefore for only 21% by the increase in multiple pregnancies typical of ART) [8].

About the effect of multiple pregnancies, Wen et al. [14] attribute to it most of the association between ART and CHD, mediated by 87%. In fact, considering this aspect, the authors reported the association as non-significant. A similar result is described in 2014 by Votava-Smith et al. [28], who pointed out that, in a logistic regression age of mother and multiple pregnancies, there was no relationship between ART and CHD. In this regard, the role of maternal age seems to be very important. In fact, Zhang et al. also assessed how the associations found between ART and CHD were non-significant after correcting for maternal age >35 years [11]. Therefore, the role of maternal age in determining the risk of CHD in ART births is more important than the role of multiple pregnancies, which are not always decisive depending on the analyzed study.

Even the diagnostic criteria used for female infertility may affect the results of a study, representing an important confounding factor. Only Fauque et al. [22] reported this issue as a risk of biases.

Another interesting issue discussed by some authors is the role of male infertility. Jwa et al. in their 2019 study found that oligozoospermia was significantly associated with a greater risk of CHD in ART pregnancies [10].

In this sense, although published studies concerning the role of de novo mutations (DNMs) associated with CHDs are limited, Wang et al. [13] suggested a notable issue. Indeed, it is well known that ART procedures increase the number of germlines DNMs in naturally conceived children for 4.59 times, even after correcting for confounding factors. In ART children, it was seen that the accumulation of non-conducting functional mutations was independently associated with CHDs and 87.9% of the mutations originated from the father. Furthermore, paternal infertility alone is associated with an increase in gDNMs in ART offspring. This indicates that indeed ART itself may not be a major reason for the accumulation of gDNMs. If this molecular aspect should be confirmed with further studies, it could explain the reason for the link between paternal infertility and CHDs.

Although published studies are limited, there is basic evidence suggesting that the underlying pathologies associated with couple infertility, mainly on the father’s side, may mediate part of the effect of the associations reported by the authors between ART and CHD and certainly deserve the attention of future research groups.

This systematic review has several limitations to consider. Firstly, most of the included papers, although judged with low risk of bias, were retrospective cohort analyses, which are, by stance, subjected to several biases and related to significant study heterogeneity in pooled results from meta-analysis. In addition, to increase the available amount of data, we decided not to exclude papers based on their quality. An additional limitation should be addressed to the lack of maternal outcomes in the included studies. However, several point of strengths should be remarked. First, the high number of studies and subjects included in the quantitative analysis. Secondly, although retrospective analyses, included papers were all classified within higher ranks in the quality assessment, reducing the overall risk of bias for interpreting the conclusions of the systematic review. Third, due to the nature of the investigated pathologies, randomized controlled trials are unperformable, and observational studies should be considered the only sources of available evidence.

## Conclusion

This systematic review and meta-analysis highlighted that, although conflicting results emerged for most topics from the data extraction, some final considerations should be considered. Considering the overall presence of CHDs, a small risk of CHDs seems retrievable in ART pregnancies when compared to spontaneous pregnancies. However, considering the pathologies by their subtype (thus distinguishing major and minor according to the need for surgical correction), there is a considerable increase of minor heart defects among births after ART procedure. These findings, such as pervious ductus arteriosus, atrial, and ventricular septal defects, often self-resolve after birth and do not require surgical interventions. For this reason, this increase should not be considered clinically significant.

About the correlation between ART and major heart defects, conflicting results emerged, requiring further investigation in the future with specifically designed studies (preferably with case-control studies, given the rarity of the pathologies). Regarding the role of confounding factors, mainly mother’s age and male infertility, several authors reported their role in the correlations between ART and CHD, whereas the role of twin pregnancies is less clear, given the discordance between the results of the studies.

Future studies should focus on inner issues of infertility rather than ART itself, in order to deeply highlight any plausible relation of such factors and CHDs. As far as has been outlined in this systematic review, at the current state of research, there are no prerequisites for proposing ultrasonographic cardiac screening in children born from ART, thus favoring a reassuring attitude towards infertile couples.

### Supplementary Information

ESM 1 (DOCX 110 kb)

ESM 2 (DOC 1970 kb)

## Data Availability

All data generated or analyzed during the present study are included in the published article and its supplementary material file.

## Code Availability

Not applicable for systematic reviews.
