# Supplementary material for: Assisted Reproductive Techniques and Risk of Congenital Heart Diseases in Children: a Systematic Review and Meta-analysis
Source: Reprod Sci. 2023 May 5;30(10):2896–906. doi: 10.1007/s43032-023-01252-6 (PMC10556115; doi:10.1007/s43032-023-01252-6)
Supplement: Supplementary file 1 — (DOCX 110 kb) [file 43032_2023_1252_MOESM1_ESM.docx]

**Table S1.** Quality scores of the studies included in the meta-analysis, assessed by the Newcastle-Ottawa scale.

|  |  | **Selection** | | | | **Comparability ^a^** | **Outcome** | | | **Overall quality** |
| --- | --- | --- | --- | --- | --- | --- | --- | --- | --- | --- |
| **Author (et al); reference** | **Year of publication** | **Representativeness of the exposed cohort** | **Selection of the non-exposed cohort** | **Ascertainment of exposure** | **Demonstration that the outcome of interest was not present at the start of the study** | **Comparability of cohorts on the basis of the design or analysis** | **Assessment of the outcome** | **Was follow-up enough for outcomes to occur** | **Adequacy of follow-up of cohorts** |  |
| Morimoto [29] | 2022 | * | * | 0 | * | * | * | * | * | 7 |
| Bjorkman [34] | 2021 | * | * | * | * | * | * | * | 0 | 7 |
| Fauque [38] | 2021 | 0 | * | * | * | * | * | 0 | * | 6 |
| Galdini [25] | 2021 | * | * | * | * | * | 0 | * | * | 7 |
| Norrman [37] | 2021 | * | * | 0 | * | ** | * | * | * | 8 |
| Serafin [31] | 2021 | * | 0 | * | * | * | * | * | * | 7 |
| Wang [41] | 2021 | * | * | 0 | * | ** | * | * | * | 8 |
| Zhang [36] | 2021 | * | * | * | * | ** | 0 | * | * | 8 |
| Aderibigbe [39] | 2020 | * | * | 0 | * | * | 0 | * | * | 6 |
| Wen [19] | 2020 | * | * | 0 | * | * | * | * | * | 7 |
| Jwa [40] | 2019 | * | * | 0 | * | ** | * | * | * | 8 |
| Pavlicek [23] | 2019 | * | * | 0 | * | ** | 0 | * | * | 7 |
| Patil [20] | 2018 | * | * | 0 | * | ** | 0 | * | * | 7 |
| Shamshirsaz [30] | 2018 | * | * | 0 | * | ** | * | * | * | 8 |
| Shechter-Maor [24] | 2018 | * | * | 0 | * | ** | 0 | * | * | 7 |
| Iwashima [21] | 2017 | * | * | * | * | ** | 0 | * | * | 8 |
| Schofield [33] | 2017 | * | * | 0 | * | ** | 0 | * | * | 7 |
| Yang [42] | 2017 | * | * | 0 | * | ** | 0 | * | * | 7 |
| Panagiotopoulou [22] | 2016 | * | * | 0 | * | * | 0 | * | * | 6 |
| Heisey [32] | 2015 | * | * | 0 | * | ** | * | * | * | 8 |
| Tararbit [28] | 2014 | * | * | * | * | ** | 0 | * | * | 8 |
| Votava-Smith [35] | 2014 | * | * | 0 | * | ** | 0 | * | * | 7 |
| Tararbit [27] | 2013 | * | * | * | * | * | * | * | * | 8 |
| Tararbit [26] | 2011 | * | * | * | * | * | * | * | * | 8 |

^a^ Comparability of cohorts: for the most important factor: study controls adjusted for age; additional factor: study controls for BMI
